# PSYCHOSOCIAL WELL-BEING, RELATIONSHIP STATUS, SEXUALITY, AND GENDER IN THE CANADIAN LONGITUDINAL STUDY ON AGING

**DOI:** 10.1093/geroni/igae098.2486

**Published:** 2024-12-31

**Authors:** Arne Stinchcombe, Douglas Hanes

**Affiliations:** University of Ottawa, Ottawa, Ontario, Canada; Stony Brook University School of Medicine, Stony Brook, New York, United States

## Abstract

Social connection and support are vital components of later-life health; for many older people, much of this support comes from a spouse or partner. Existing research has shown that gender and sexuality are central to the effects of partnership, and thus the later-life psychosocial and mental health benefits of partnership also likely differ by these factors. Canadian Longitudinal Study of Aging (CLSA) participants were categorized according to gender and sexuality; we analyzed how the effects of relationship status differed across these categories. Several psychosocial and mental health outcome variables were tested, including depression symptoms, mood disorder, anxiety, self-rated health, social support, and social standing. Linear and ordered logistic regressions were conducted, adjusting for age and education. Married persons had lower depression scores (B=-2.20, p<.001), higher social support (B=21.49, p<.001), and subjective status (B=.61, p<.001) than other relationship categories. The differences themselves differed by gender and sexuality: lesbian/bisexual women were better overall and experienced less adverse impact (B=23.37, p<.001), while these had greater effects on gay/bisexual men (B=21.10, p<.001). Lesbian/bisexual women have greater social supports across relationship statuses, while gay/bisexual men have less social support if not partnered. Findings highlighted variable and gendered effects of relationship status and sexual orientation on psychosocial outcomes. Examining differential effects for gender and sexual minority groups is essential to inclusive gerontological research.

